# Predicting the Potential Suitable Distribution of Tomato Brown Rugose Fruit Virus in China Using a BIOMOD2 Ensemble Model

**DOI:** 10.3390/biology15191743

**Published:** 2026-10-02

**Authors:** Linfei Shou, Yuxi Wang, Yaqian Shang, Jiaqi Lu, Min Liu, Shuxing Zhou, Jianzhong Li, Jinming Zhang

**Affiliations:** 1 Zhejiang Provincial Plant Protection, Quarantine and Pesticide Management Station, Hangzhou 310020, China; 2 State Key Laboratory for Quality and Safety of Agro-Products, Key Laboratory of Biotechnology in Plant Protection of MOA of China and Zhejiang Province, Institute of Plant Protection and Microbiology, Zhejiang Academy of Agricultural Sciences, Hangzhou 310021, China; 3 College of Life and Environmental Sciences, Hangzhou Normal University, Hangzhou 311121, China; 4 Centre of Agro-Technology and Extension of Qujiang, Quzhou 324002, China

**Keywords:** tomato brown rugose fruit virus, BIOMOD2, species distribution model, potential suitable area, CMIP6, greenhouse agriculture

## Abstract

Tomato brown rugose fruit virus (ToBRFV) is an emerging tobamovirus that threatens tomato production and seedling trade. We used a BIOMOD2 ensemble to model present and future environmental suitability in China from 35 screened occurrence records, climatic predictors, and agricultural context data. Climate projections were based on BCC-CSM2-MR under SSP126, SSP245, SSP370, and SSP585 for the 2050s and 2090s. The ensemble achieved calibration TSS = 0.831 and AUC/ROC = 0.958. Spatial-block evaluation was lower and more variable, so the calibration metrics do not demonstrate equally strong geographic transferability. Bio6 and bio15 were the leading predictors, followed by bio5 and bio13. At the primary AUCroc threshold of 0.441, the current suitable area was 44.35 × 10^4^ km^2^ (approximately 4.62% of China’s land area), concentrated in warm, humid eastern and southern regions. Future estimates depend on the selected GCM and binary threshold. The maps identify environmental suitability and monitoring priorities rather than epidemic probability.

## Simple Summary

Tomato brown rugose fruit virus can damage tomato crops and spread through contaminated seeds, seedlings, tools, and greenhouse operations. Occurrence records remain limited, which complicates the identification of surveillance priorities. We combined 35 screened occurrence records with climate and agricultural data in an ensemble of models to map areas of China that may be environmentally suitable now and under future climates. The highest current suitability occurred in warm, humid parts of eastern and southern China. Future maps retained a southern core and suggested expansion in several northern and western regions. Some areas that are currently suitable may decline under high-emission conditions. The maps should be considered together with confirmed records, tomato production, greenhouse distribution, and human-mediated movement.

## 1. Introduction

Tomato (*Solanum lycopersicum* L.) production relies on connected seed, seedling, and fruit supply chains. In many regions, it also relies on intensive protected cultivation. Disease prevention therefore affects crop yield and quality, vegetable supply, and the biosecurity of seedling trade. Tomato brown rugose fruit virus (ToBRFV; genus *Tobamovirus*) has become a major concern because it has spread through tomato production systems over the past decade. It was first described in greenhouse tomatoes in Jordan. Later work in Israel showed that it could infect tomatoes carrying the *Tm-2*^2^ resistance gene, which had previously provided resistance to tobacco mosaic virus and tomato mosaic virus [1,2]. Studies from Eurasia, North America, and the Mediterranean region have examined detection, host responses, molecular diversity, transmission, and outbreak dynamics [3,4]. Available evidence points to seed-associated and mechanical spread, resistance breaking, and rapid international movement [5]. Genomic and phylogenetic analyses provide complementary information on isolate diversity and outbreak links [6,7].

ToBRFV infection can reduce both tomato yield and marketability: foliar symptoms include mosaic, chlorosis, rugosity, and deformation, whereas fruit may develop brown lesions, rugose surfaces, or malformation [2,3]. Control is complicated because opportunities for virus movement occur throughout the production chain. Contact between plants, pruning and grafting practices, contaminated tools, worker activity, and infected seeds or seedlings can all contribute to spread. In protected production systems, pollinating insects and repeated cultivation cycles can further increase within-facility transmission opportunities [3,4,8,9]. The virus is predominantly associated with the seed coat; even where direct seed-to-seedling transmission is uncommon, contaminated seed can serve as a source for mechanical spread [10]. Its detection in irrigation water and on common greenhouse surfaces makes sanitation, water management, tool disinfection, and seedling-source control central components of prevention [11,12,13].

ToBRFV has been reported from several parts of China [14,15,16]. Domestic studies have addressed detection and mixed infection [17,18,19], resistance resources [20], and geographic suitability modeling [21]. Available diagnostic methods include validated RT-PCR, real-time RT-PCR, ddPCR, nanopore sequencing, and immunological tests [22,23,24,25,26]. Resistance research has mapped relevant loci, examined wild germplasm, tested *TOM1* editing, and identified tissue-dependent responses [27,28,29,30]. Resistance-breaking isolates and complex tobamovirus–host recognition nevertheless require continued surveillance [31,32,33]. In the present compiled dataset, occurrence records were limited and spatially clustered. Climate, host exposure, protected cultivation, and movement of infected materials can jointly affect virus occurrence. Known records alone cannot define early warning priorities; modeled suitability instead helps identify areas that warrant surveillance and management attention.

Species distribution models (SDMs) relate recorded occurrences to environmental conditions to estimate where a focal organism may find suitable conditions. They are widely used in spatial assessments of invasive organisms, crop pests, and plant diseases [34,35]. BIOMOD2 combines several statistical and machine learning algorithms. Its ensemble approach can reduce reliance on the assumptions of a single algorithm and provide more stable spatial estimates [36,37,38]. For occurrence-based SDMs, inference is limited by sparse and geographically biased records, uneven sampling, and incomplete representation of human-mediated movement [34,39,40,41,42]. For ToBRFV, the model therefore serves as a spatial tool for prioritizing surveillance, not as a direct measure of epidemic intensity.

Changes in temperature, precipitation, and extreme events can alter crop environments and shift the potential distributions of pests and other organisms [43,44,45,46]. Climate-only models may miss processes linked to host availability and production practices [42,47]. We evaluated tomato-host distribution and greenhouse-facility coverage alongside bio-climatic variables; greenhouse-facility coverage was retained in the final BIOMOD2 ensemble, whereas tomato-host distribution was assessed in sensitivity analyses. We estimated current and future ToBRFV suitability in China under CMIP6 scenarios, examined influential predictors and spatial patterns, and quantified changes in suitable area. The results are intended to support surveillance design, seed and seedling quarantine, and management in protected tomato production.

## 2. Materials and Methods

### 2.1. Occurrence Data Collection and Screening

We compiled ToBRFV occurrence coordinates mainly from GBIF (https://www.gbif.org/) and checked them against published occurrence reports (Figure 1). We standardized taxonomic names and coordinate formats, then excluded records with missing coordinates, obvious coordinate errors, duplicated locations, or unsuccessful environmental value extraction. The final dataset contained 35 valid records, with longitudes ranging from 81.199929 to 121.115080 degrees E and latitudes ranging from 25.635000 to 43.831386 degrees N. The occurrence records did not include reliable source-year information, so temporal coverage cannot be reported. No exact duplicate coordinates remained. Although a 10 km spatial filter was applied, 16 records fell within the same 0.2-degree grid cell. BIOMOD2 may further remove records that share a raster cell. We did not apply target-group or sampling effort bias correction; this limitation is considered when interpreting the model.

### 2.2. Environmental Variable Selection

The initial predictor pool contained 19 bio-climatic variables and two agricultural variables (Table 1). The final BIOMOD2 configuration retained bio3, bio5, bio6, bio8, bio13, bio15, and greenhouse_facility_fraction (hereafter bio21). Current bio-climatic layers came from WorldClim v2.1 at 2.5 arc-min resolution and represented the 1970–2000 baseline [48]. Future layers came from BCC-CSM2-MR for SSP126, SSP245, SSP370, and SSP585 in 2041–2060 and 2081–2100 [49,50]. The scenario comparison therefore describes differences among pathways and time slices within one GCM; it does not measure uncertainty among climate models.

To represent agricultural context, we used a tomato-host layer and a greenhouse-facility layer for China. Tomato harvested area from SPAM2020 V2r2 was used as a proxy for host planting intensity [51]. Greenhouse coverage was calculated from the 2022 national greenhouse distribution raster [52]. All layers were cropped to China and aligned to a common projection, resolution, and extent. The tomato layer was bilinearly resampled to the climate grid; greenhouse area was aggregated within each target cell and expressed as a 0–1 coverage fraction. Only climate layers varied in future projections. Tomato-host distribution and greenhouse coverage were held at their present spatial patterns.

We evaluated collinearity using Pearson correlations and variance inflation factors (VIFs) calculated from values at occurrence locations. For the original model, pairs were screened using |r| < 0.8 and multivariable inflation using VIF < 10, following commonly used collinearity-screening practice [53]. The final predictor set retained bio3, bio5, bio6, bio8, bio13, bio15, and bio21 (Table 2). To evaluate sensitivity to the Pearson criterion, we conducted two complete reruns under the same China-mask training and projection domain, using |r| < 0.7 and |r| < 0.8 while holding VIF < 10 and the 35 occurrence records, 21 candidate layers, pseudo-absence and cross-validation settings, algorithms, ensemble settings, seed, and projection settings constant. The |r| < 0.7 rerun retained bio3, bio5, bio6, bio8, bio13, bio15, and bio21 after Pearson screening; the |r| < 0.8 rerun retained the same variables plus bio14. After VIF screening, both reruns retained the same final set: bio3, bio5, bio6, bio8, bio13, bio15, and bio21. The paired binary current maps agreed for 97.88% of 24,287 valid China-mask cells, although their internally derived AUCroc thresholds and suitable-area estimates differed. These results are reported as a supplementary predictor-screening sensitivity analysis and do not replace the original model estimates. We also compared climate-only, climate-plus-tomato-host, climate-plus-greenhouse, and climate-plus-both specifications as sensitivity analyses. These comparisons were intended to describe the incremental contribution to model prediction, not to establish a causal biological mechanism. The main projection configuration was chosen on the combined basis of collinearity, model settings, and interpretability.

### 2.3. Construction of Species Distribution Models

We fitted current and future suitability models with the biomod2 R package (version 4.2-6-2; https://biomodhub.github.io/biomod2/) in R 4.4.3 (R Core Team, R Foundation for Statistical Computing, Vienna, Austria) [37]. The candidate algorithms were GLM, GBM, GAM, CTA, ANN, FDA, SRE, MARS, RF, and MaxEnt. Their full names are provided above. MaxEnt was included because it is designed for prediction from presence and background data [54].

For each random evaluation, 80% of the occurrence records were used for calibration and 20% for testing; this split was repeated four times. Since presence-only data were available, BIOMOD2 generated pseudo-absences at random, following established pseudo-absence selection principles [55]: three sets of 2000 points were created with prevalence fixed at 0.5, and each used the same 80/20 resampling design. The pseudo-absence/background sampling was neither accessibility weighted nor target-group corrected. We additionally ran spatial-block validation across 27 combinations, defined by three pseudo-absence sets and nine blocks; only 18 occurrence records were represented across those blocks. This complementary spatial assessment is informative but uncertain because block sizes were small and uneven. Single models with validation TSS ≥ 0.6 entered the ensemble, which used TSS-weighted means (EMwmean) calculated from validation results.

We assessed discrimination with AUC and TSS. AUC summarizes separation of presence from absence or background observations [56], whereas TSS combines sensitivity and specificity while being less sensitive to prevalence [57]. Models meeting the validation TSS ≥ 0.6 criterion contributed to the EMwmean ensemble in proportion to their TSS. The resulting weighted prediction is a suitability score on a 0–1 scale.

### 2.4. Visualization and Spatial Change Analysis

For descriptive maps, we used five suitability classes: unsuitable (0 ≤ *p* < 0.2), low (0.2 ≤ *p* < 0.4), moderate (0.4 ≤ *p* < 0.6), high (0.6 ≤ *p* < 0.8), and extremely high (0.8 ≤ *p* ≤ 1). For the original model, the ensemble evaluation yielded an AUCroc cutoff of 441 on BIOMOD2’s 0–1000 prediction scale (0.441 on the 0–1 scale); the pixel-level verification of this cutoff is provided in Supplementary Table S3. We used 0.441 as the primary threshold for original model area and change estimates. To evaluate binary-threshold sensitivity, we additionally reclassified the retained continuous current and future ensemble rasters at 0.391, 0.421, 0.461, 0.491, 0.500, 0.510, and 0.515. Suitable area, contraction, expansion, stable area, loss rate, gain rate, and signed net range-change rate at each threshold are reported in Supplementary Table S1.

For spatial change calculations, cells suitable at present but unsuitable in a future scenario were counted as contractions, newly suitable cells as expansions, and cells suitable in both periods as stable. Let C, F, S, L, and E denote current suitable area, future suitable area, stable area, contraction area, and expansion area, respectively. We calculated F = S + E and C = S + L. The loss rate was 100 × L/C, the expansion rate was 100 × E/C, and the signed net range-change rate was 100 × (E − L)/C. Areas were calculated as class cell count divided by the 24,287 valid current binary grid cells (NoData excluded), multiplied by China’s land area (960 × 10^4^ km^2^).

## 3. Results

### 3.1. Model Accuracy and Importance of Environmental Variables

Across 10 algorithms, three pseudo-absence sets, and four repeated random runs, we obtained 108 valid single-model evaluations after excluding failed or invalid runs. The final EMwmeanByTSS ensemble had calibration TSS = 0.831 and AUC/ROC = 0.958. Calibration sensitivity was 94.444%, and specificity ranged from 88.647% to 88.738%. These values describe the random calibration/evaluation workflow. The spatial-block assessment comprised 27 model combinations. Mean spatial-block AUC ranged from 0.544 for ANN to 0.759 for MaxEnt, and mean spatial-block TSS ranged from −0.002 for GBM to 0.355 for SRE. Most mean TSS values were near zero. The random-evaluation metrics should not be taken as evidence of uniformly strong geographic extrapolation.

Pearson correlation analysis showed varying degrees of correlation among candidate environmental variables (Figure 2). The effective final model used bio3, bio5, bio6, bio8, bio13, bio15, and greenhouse_facility_fraction. Variable importance analysis ranked minimum temperature of the coldest month (bio6) highest, followed by precipitation seasonality (bio15), maximum temperature of the warmest month (bio5), and precipitation of the wettest month (bio13) (Figure 3). In separate sensitivity analyses, climate-only, climate-plus-tomato-host, climate-plus-greenhouse, and climate-plus-both schemes were fitted with the climate predictors held constant. In the smaller direct host/greenhouse comparison, the GLM climate-only scheme had mean AUC/TSS values of 0.813/0.450, the climate-plus-tomato-host scheme 0.733/0.467, the climate-plus-greenhouse scheme 0.653/0.200, and the climate-plus-both scheme 0.900/0.617 (n = 2 repeated evaluations). The larger covariate comparison using three pseudo-absence replicates and four random repeats also showed algorithm-dependent responses: adding tomato_host improved AUC for several algorithms, whereas adding greenhouse_facility_fraction or all extras was less consistent. These results support reporting host and greenhouse variables as sensitivity factors, not universal causal drivers.

Response curves for the seven retained predictors (bio3, bio5, bio6, bio8, bio13, bio15, and greenhouse_facility_fraction) were examined to complement variable importance (Figure 4). The curves show predictor-specific, nonlinear changes in model suitability across environmental gradients and are interpreted as model response patterns rather than causal physiological relationships. In particular, the response curve analysis was used to identify the environmental ranges associated with higher or lower predicted suitability and to avoid interpreting variable importance rankings alone as biological causation.

### 3.2. Current Potential Suitable Areas of ToBRFV

The ensemble model predicted that potentially suitable areas for ToBRFV under current climatic conditions were mainly distributed in eastern and southern China (Figure 5). Continuous suitability maps showed that highly and extremely suitable areas were concentrated from southern East China to eastern South China, including Jiangxi, Fujian, southern and eastern coastal Zhejiang, northern Guangdong, and Taiwan. Moderately suitable areas were mainly distributed around Hunan, Guangxi, Guangdong, Fujian, and Zhejiang, with local extensions into southwestern China. Low-suitability areas occurred as patches in central China, parts of northern China, northern Xinjiang, some mountainous areas of southwestern China, and southern northeastern China. The arid northwest, most of the Qinghai–Tibet Plateau, Inner Mongolia, and northern northeastern China showed overall low suitability.

Using the primary AUCroc threshold of 0.441, the current suitable area of ToBRFV in China was 44.35 × 10^4^ km^2^, accounting for approximately 4.62% of the national land area. This value was used as the current-area baseline for the area-change analysis in Table 3. The threshold definitions and binary classification rule are reported in the Section 2. Current suitable areas were mainly located in eastern and southern China. Their overlap with tomato production and protected vegetable cultivation is considered for surveillance planning, but the model does not establish a direct causal effect of agricultural intensity.

### 3.3. Future Changes in Potential Suitable Areas Under Different Climate Scenarios

Climate scenarios produced different suitability patterns across future time slices (Figure 6). Using the current suitable-area baseline of 44.35 × 10^4^ km^2^, the BCC-CSM2-MR projections varied in both magnitude and spatial pattern among SSP pathways and periods. They are based on one GCM, the selected binary threshold, and static non-climatic layers. They do not fully quantify climate model or agricultural change uncertainty.

Using the primary AUCroc threshold of 0.441, projected future suitable area and range change differed among pathways and periods (Table 3). Under SSP126, total suitable area was 53.84 × 10^4^ km^2^ in the 2050s and 53.01 × 10^4^ km^2^ in the 2090s; signed net range-change rates were +21.39% and +19.52%, respectively. Under SSP245, total suitable area was close to the current baseline (44.27 × 10^4^ km^2^ in the 2050s and 44.63 × 10^4^ km^2^ in the 2090s), with signed net changes of −0.18% and +0.62%. Under SSP370, total suitable area was 43.08 × 10^4^ km^2^ in the 2050s and 50.16 × 10^4^ km^2^ in the 2090s, corresponding to −2.85% and +13.10% signed net change.

Under SSP585, contraction was more pronounced. Total suitable area was 27.51 × 10^4^ km^2^ in the 2050s and 25.42 × 10^4^ km^2^ in the 2090s. Contraction covered 25.10 and 29.88 × 10^4^ km^2^, with loss rates of 56.60% and 67.38%, respectively; signed net range-change rates were −37.97% and −42.69%. These values are conditional on the selected binary threshold. The threshold sensitivity analysis showed that absolute area estimates and, for some scenario–period combinations, the sign of net range change changed across thresholds. We therefore interpret the maps as identifying a persistent spatial suitability core and threshold-sensitive marginal changes, rather than claiming uniform expansion under all scenarios.

Future spatial-change maps showed that stable suitable areas were mainly distributed around the core belt from Jiangnan to South China, especially in Jiangxi, Fujian, northern Guangdong, southern Hunan, and adjacent regions. Expansion areas appeared as patches under different scenarios and were mainly located in northern China, the Shandong Peninsula, southern Liaoning, northern Xinjiang, and some southwestern regions. Contraction areas were mostly located along the margins of current highly suitable core areas and expanded inward under the high-emission SSP585 scenario.

Future suitability showed a relatively stable core, patchy threshold-sensitive changes at the periphery, and greater local contraction under high emissions. Surveillance and control should focus on the Jiangnan–South China core and on potential change patches in northern China, Shandong, southern Liaoning, and northern Xinjiang. Tomato-host distribution and greenhouse coverage should be considered when setting these priorities.

Supplementary Table S1 reports the full binary-threshold sensitivity analysis (eight thresholds, all scenario–period combinations), and Supplementary Table S2 reports the matched Pearson cutoff sensitivity reruns.

## 4. Discussion

### 4.1. Model Reliability and Interpretation of Key Variables

This study constructed a BIOMOD2 ensemble model based on 10 single algorithms. The final ensemble showed calibration TSS = 0.831 and AUC/ROC = 0.958 under the random-evaluation workflow. However, the complementary 27-combination spatial-block assessment showed lower and highly variable geographic performance: mean AUC ranged from 0.544 to 0.759 and mean TSS ranged from −0.002 to 0.355 among algorithms. This contrast is consistent with broader SDM evidence showing that random-split metrics can overstate geographic transferability when occurrence data are spatially dependent [41]. The recent China-focused MaxEnt study by Zheng et al. provides a useful methodological comparator for ToBRFV mapping in China [21]. However, its occurrence compilation, predictor selection, algorithm, and evaluation design differ from those used here, so numerical performance values and binary areas should not be compared directly. Our spatial-block results therefore provide a conservative indication of geographic uncertainty within the present modeling design. AUC and TSS should be interpreted within the evaluation design rather than as universal evidence of predictive reliability [56,57].

In the final ensemble, bio6 and bio15 had the strongest associations with predicted ToBRFV suitability, followed by bio5 and bio13. This differs from the China MaxEnt analysis by Zheng et al., which emphasized bio9, bio8, and bio4 [21]. Such differences are expected because predictor rankings depend on occurrence records, accessible area, collinearity screening, spatial resolution, and model algorithm; they should not be interpreted as contradictory physiological thresholds. Both studies nevertheless identify climate as a primary component of the modeled environmental niche. We also compared host and greenhouse covariates. In the direct GLM comparison, tomato_host alone did not improve mean AUC over the climate-only model, whereas greenhouse alone reduced mean AUC. Adding both variables increased mean AUC to 0.900 and mean TSS to 0.617. This comparison used only two repeated evaluations and is exploratory. The larger multi-algorithm comparison also produced algorithm-dependent effects. This result accords with the commercial greenhouse survey by Di Rosa et al., which found no association between the measured greenhouse features and ToBRFV diagnosis [12]. Response curves and covariate comparisons therefore describe model associations and sensitivity, not biological causation. Seed contamination and mechanical transmission are established components of ToBRFV epidemiology [10]. Survival in water provides a separate rationale for attention to water management [11]. The persistence of ToBRFV on common glasshouse surfaces supports rigorous sanitation and tool disinfection in protected production [13]. Resistance breeding and resistance gene engineering remain complementary management approaches [58,59]. A recent screening of 476 accessions also identified resistant or tolerant material in multiple *Solanum* species, including highly resistant *S. pimpinellifolium* lines [29]. However, resistance deployment should be combined with viral surveillance and hygiene: a single movement protein amino acid substitution was sufficient to break a newly deployed ToBRFV resistance in a commercial greenhouse setting [31].

### 4.2. Current and Future Suitability Patterns and Implications for Management

Current projections indicated that the potential suitable areas of ToBRFV are mainly distributed in eastern and southern China. This broad eastern/southern emphasis is broadly comparable with the China MaxEnt study by Zheng et al. [21], although exact area estimates and local boundaries should not be compared directly because the two studies used different occurrence data, predictors, algorithms, thresholds, and validation designs. These are climate-associated suitability patterns, not direct evidence that greenhouse production caused the predicted distribution. For management, we distinguish four evidence layers: confirmed occurrence records, climate-associated suitability, tomato production/host availability, and greenhouse or human-mediated transmission pathways. ToBRFV can be disseminated through infected seed and mechanical contamination [10]. In commercial greenhouses in Quebec, ToBRFV-positive samples were detected across facilities without an association with the assessed greenhouse features, reinforcing the need not to equate greenhouse presence with infection probability [12]. This is consistent with our sensitivity analysis, in which greenhouse coverage was not a universal performance-improving predictor across algorithms. In contrast, an SDM estimates environmental association rather than a transmission pathway [35]. In high-suitability production regions, this distinction supports operational hygiene measures in addition to surveillance: experiments have shown that appropriate agricultural disinfectants can substantially reduce infectivity on contaminated worker clothing [60] and can inactivate ToBRFV in greenhouse production contexts [61]. Jiangxi, Fujian, southern Zhejiang, northern Guangdong, southern Hunan, and adjacent regions are therefore described as climate-suitability priorities, while confirmed occurrences and production–trade corridors require separate surveillance attention.

Under the BCC-CSM2-MR scenarios, the 0.441 primary threshold estimates varied by pathway and period: SSP126 showed positive signed net range change, SSP245 remained close to the current baseline, SSP370 shifted from a small 2050s contraction to a 2090s expansion, and SSP585 showed marked contraction. Binary-threshold sensitivity showed that absolute suitable-area estimates and some marginal net range-change directions depend on the cutoff. The results therefore support a persistent eastern/southern suitability core, whereas peripheral expansion and contraction estimates should be interpreted conditionally on the selected threshold. This threshold dependence is especially important when comparing our results with the published China MaxEnt projection because a change in cutoff can alter binary area estimates even when continuous suitability patterns are broadly similar [21]. Use of one GCM does not capture the full range of climate model uncertainty in distribution forecasts [46]. More broadly, SDM structural uncertainty can be substantial and should be quantified alongside climate scenario uncertainty [62]. Holding host and greenhouse layers constant also omits potentially important changes in host availability [47].

Low-suitability areas may still contain confirmed occurrences or support transmission. Among the occurrence records, one was located in Shandong and none in Anhui. Anhui is therefore not included as a quantitatively represented regional record. The Shandong occurrence had a current ensemble score of 602 on the BIOMOD2 0–1000 scale (approximately 0.602). This score was above the primary AUCroc cutoff of 441 (0.441), but this single mapped occurrence is not an independent validation result. Sparse and spatially biased occurrence data can produce mismatches between records and broad modeled suitability [39,40]. Host distribution, greenhouse production, and movement of contaminated seed or seedlings may provide additional explanations because these processes are not represented as dynamic transmission data in the present model [10,12].

### 4.3. Limitations and Future Perspectives

This study has several limitations. First, the 35 occurrence records were derived mainly from public databases and the literature, lacked reliable source-year information, and were subject to spatial clustering. A 10 km spatial filter was applied, but no target-group or effort-based sampling bias correction was implemented. Spatial thinning can reduce clustering but does not by itself resolve all sources of occurrence data bias [39]. Positional uncertainty can also affect SDM inference [40]. Pseudo-absence selection is another methodological choice that can influence model results [55]. Second, although a complementary 27-combination spatial-block assessment was conducted, the nine blocks contained only 18 occurrence points in total and had uneven presence counts; spatial metrics were therefore variable and uncertain. Third, we conducted a matched Pearson cutoff sensitivity analysis (|r| < 0.7 versus |r| < 0.8) while holding VIF < 10 and the remaining modeling settings constant. This analysis showed stability of the final VIF-screened predictor identity but did not evaluate alternative VIF cutoffs; further work should therefore assess a broader range of collinearity screening choices. Fourth, future climate projections used only BCC-CSM2-MR, and future tomato-host and greenhouse layers were held constant rather than dynamically projected. This design does not characterize structural or inter-GCM uncertainty, both of which can widen with longer forecast horizons and environmental extrapolation [62]. Fifth, source-year metadata were unavailable for the occurrence records, so temporal coverage could not be analyzed. Sixth, a quantitative integrated risk prioritization index combining climate suitability, confirmed occurrence, tomato production, greenhouse distribution, and human-mediated transmission was not constructed; these components are therefore presented as separate evidence layers. Finally, the model did not incorporate seedling propagation, fruit transport, quarantine interceptions, cultivation management, cultivar resistance, or other process variables that directly affect transmission. Consequently, the results should be used as conditional environmental suitability evidence for monitoring and management prioritization, rather than direct estimates of epidemic probability or quarantine risk.

Future work should integrate systematic monitoring sites, source-year metadata, target-group or effort-based bias correction, and spatially independent validation with better-balanced blocks. Better occurrence data design and transparent reporting are central to reliable SDM inference [34]. Pseudo-absence strategies should be evaluated explicitly during model development [55]. Spatially independent validation is needed before high random-evaluation scores are interpreted as geographic transferability [41]. Comparative work should also test whether the different predictor rankings reported by BIOMOD2 and MaxEnt analyses of ToBRFV in China persist when both frameworks use an identical occurrence dataset, predictor pool, accessible area, and validation design [21]. Future projections should incorporate multiple CMIP6 GCMs because uncertainty should be measured and communicated in distribution forecasts [46,62]. Dynamic host and greenhouse layers should be considered because host availability can alter projected risk areas [47]. Finally, field epidemiological datasets are needed to connect climate-associated suitability with infection occurrence, because the commercial greenhouse survey by Di Rosa et al. did not identify a simple greenhouse-feature profile associated with ToBRFV diagnosis [12]. A reproducible prioritization framework could first report climate-associated suitability, confirmed occurrences, tomato production, greenhouse distribution, and human-mediated transmission as separate layers before combining them. These steps would improve uncertainty assessment and clarify how environmental suitability relates to epidemic risk.

## 5. Conclusions

This BIOMOD2 ensemble mapped current and future ToBRFV suitability in China. Using the primary AUCroc threshold of 0.441, the current suitable-area baseline was 44.35 × 10^4^ km^2^ and was used for the primary area-change analysis. The threshold sensitivity analysis showed that absolute area estimates and some marginal range-change directions depend on the binary cutoff; consequently, the results support a persistent eastern/southern suitability core but should not be interpreted as threshold-invariant estimates of peripheral expansion or contraction. The final model retained seven predictors: six bio-climatic variables and greenhouse-facility coverage. Bio6 and bio15 made the largest contributions. Random-evaluation metrics were high (calibration TSS = 0.831; AUC/ROC = 0.958), whereas spatial-block performance was lower and variable across 27 combinations. The maps should therefore be applied cautiously outside the random-evaluation setting. Response curves and host/greenhouse sensitivity analyses aided interpretation. More robust projections will require multiple GCMs, dynamic agricultural layers, temporal occurrence metadata, explicit bias correction, broader collinearity screening, and an integrated prioritization index.

## Figures and Tables

**Figure 1:**
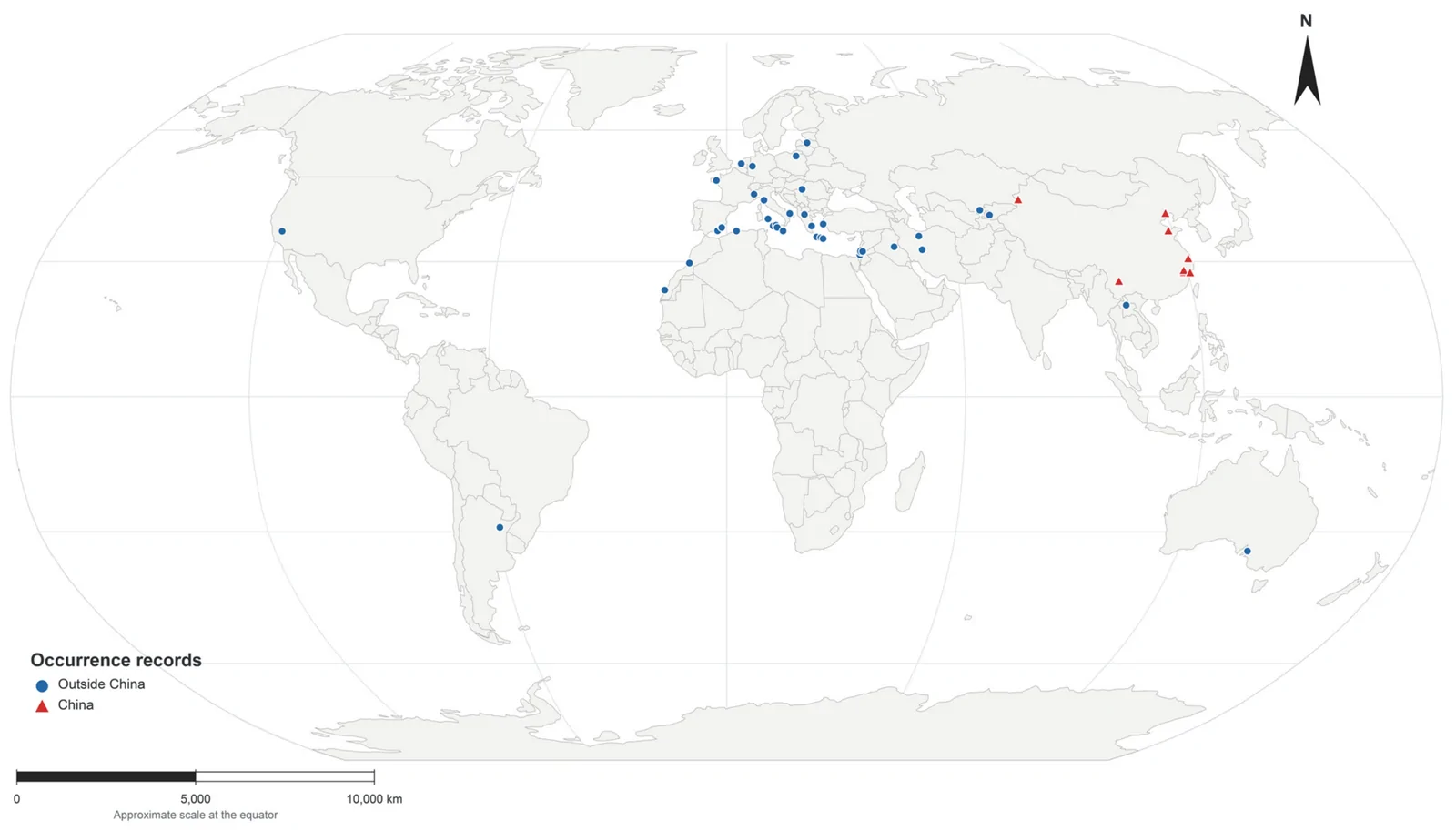
Global occurrence records of ToBRFV used for distributional interpretation. Chinese occurrence records are distinguished from records outside China. The base map was obtained from the Standard Map Service of the Ministry of Natural Resources of the People’s Republic of China, with map approval number GS(2016)1560. The map is used only for scientific illustration, and the boundary representation does not imply any political or administrative position of the authors or publisher.

**Figure 2:**
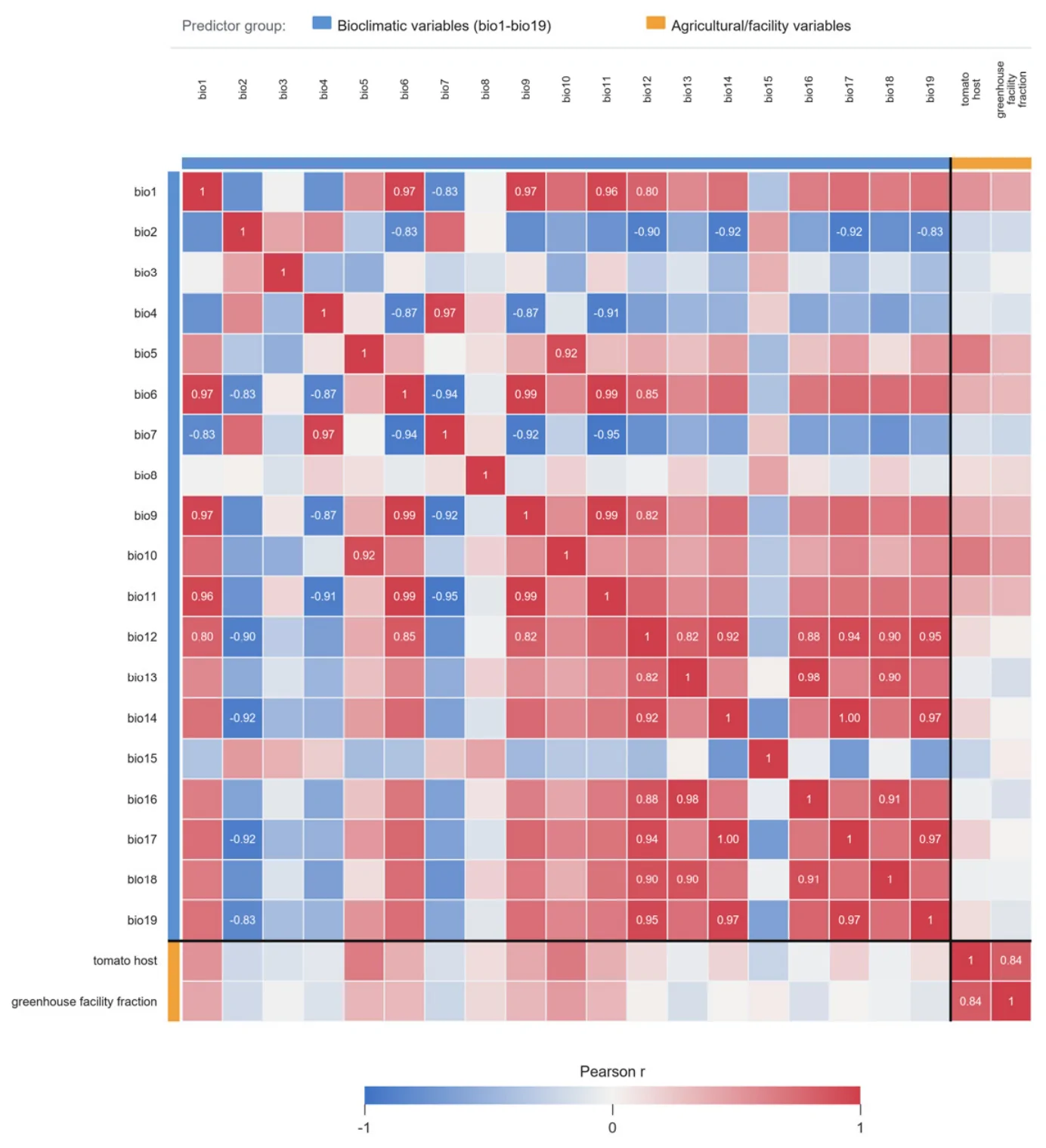
Heatmap of Pearson correlation coefficients (r) among candidate environmental variables.

**Figure 3:**
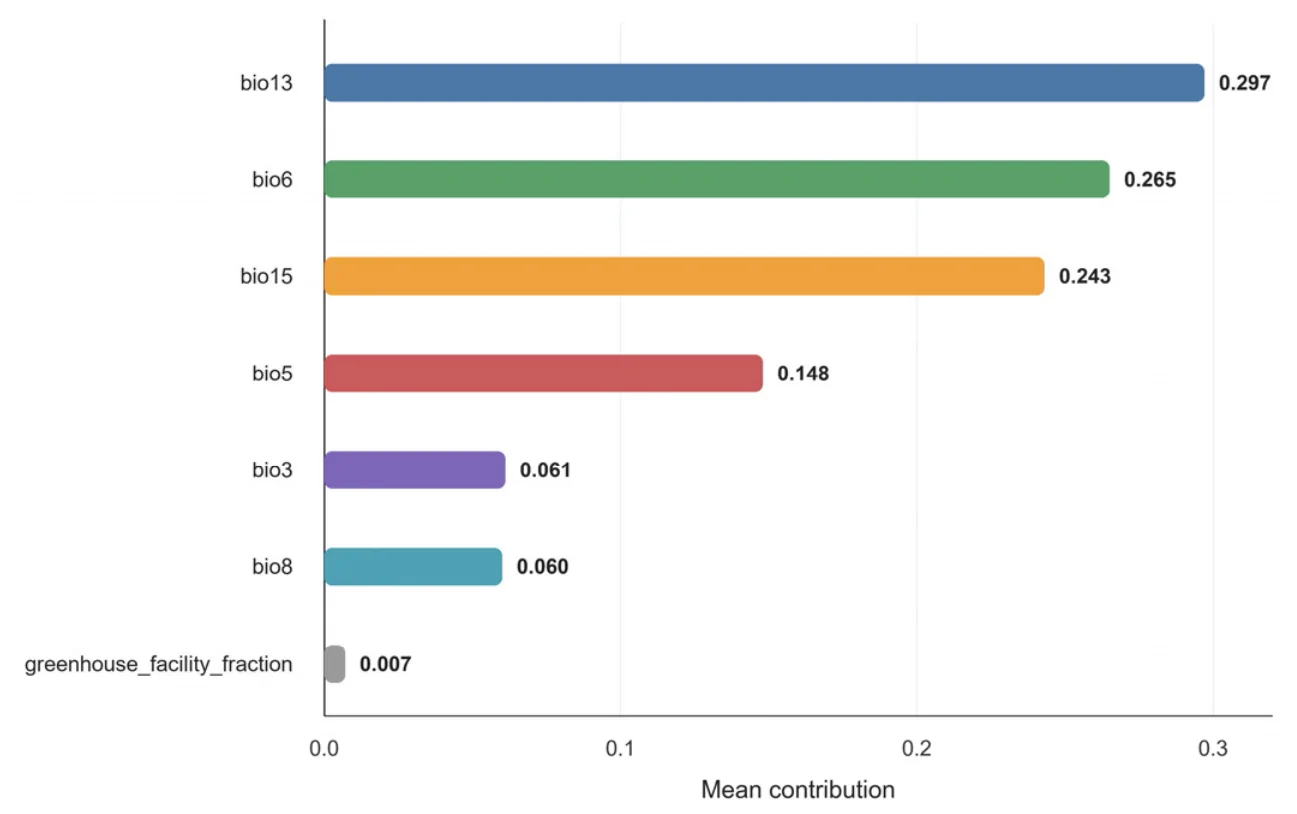
Environmental variable importance in the ensemble model.

**Figure 4:**
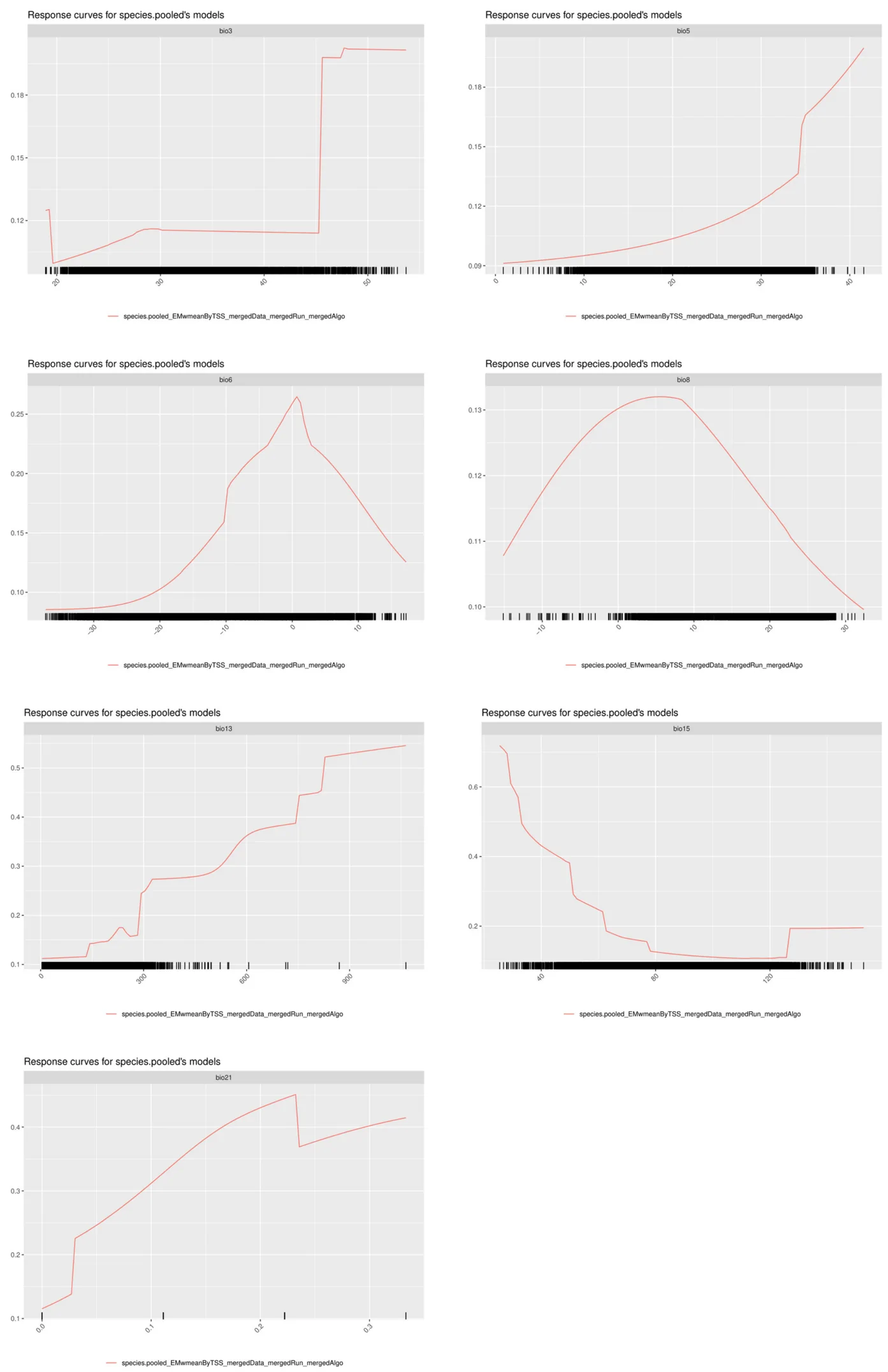
Response curves for the seven predictors retained in the final BIOMOD2 ensemble model (bio3, bio5, bio6, bio8, bio13, bio15, and greenhouse_facility_fraction). Curves show model-predicted suitability across each environmental gradient and are interpreted as response patterns rather than direct evidence of biological causation.

**Figure 5:**
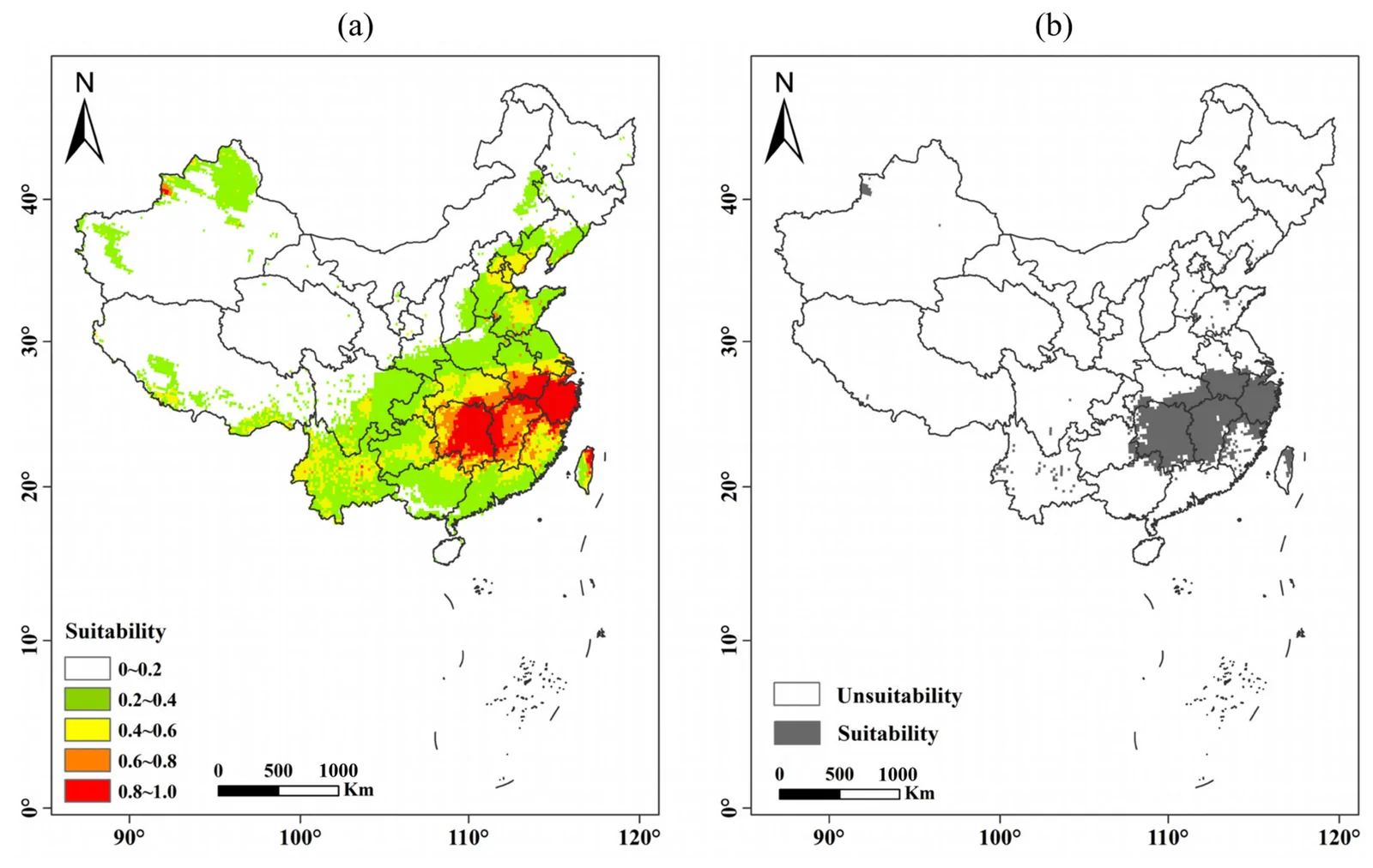
Current potential suitable distribution of ToBRFV: (**a**) continuous suitability and (**b**) binary suitable/unsuitable distribution. This figure was prepared based on the standard map with approval number GS(2020)4619, obtained from the Standard Map Service of the Ministry of Natural Resources of the People’s Republic of China. The base map was not modified. The map is used only for scientific illustration, and the boundary representation does not imply any political or administrative position of the authors or publisher.

**Figure 6:**
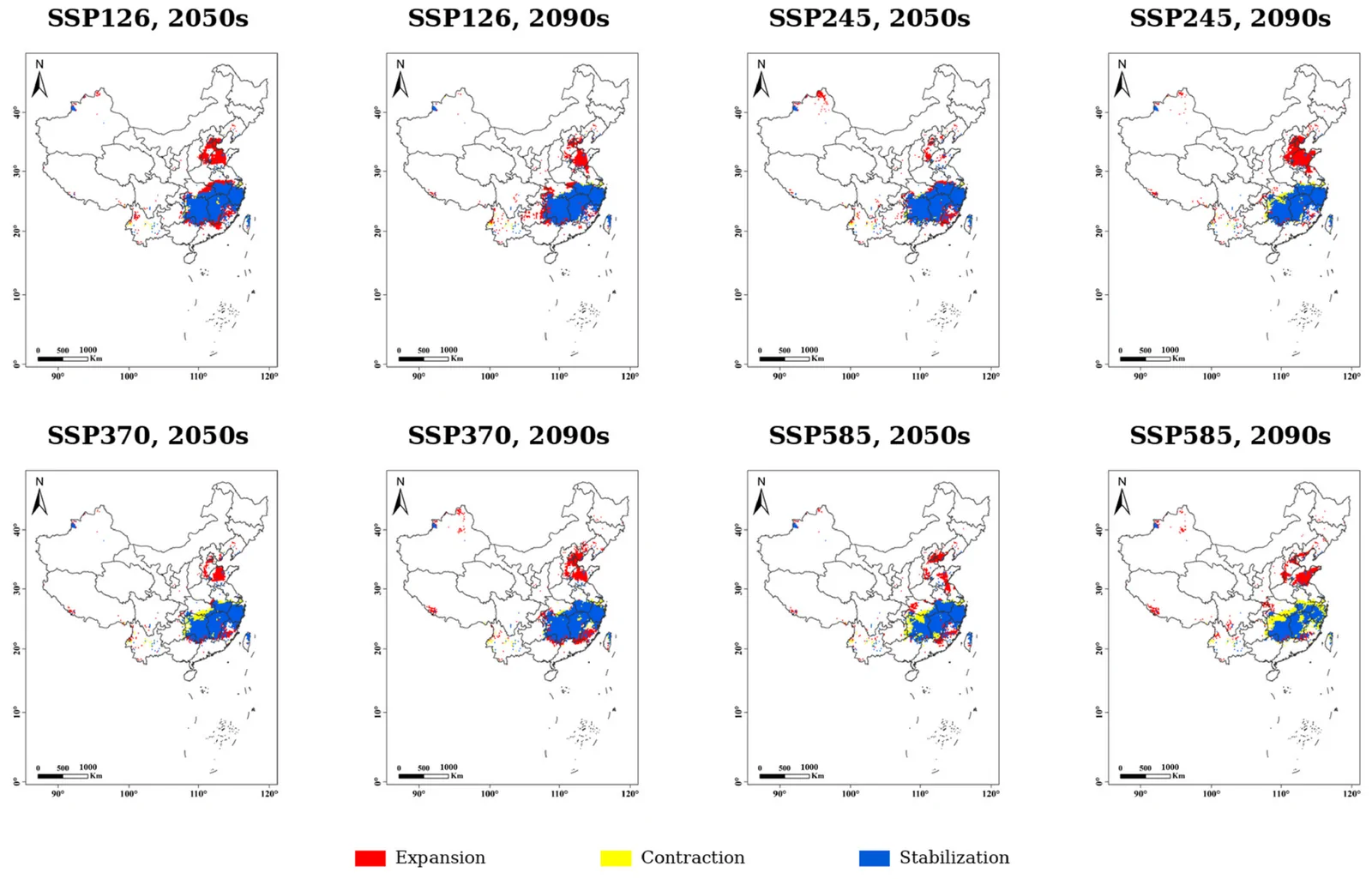
Spatial change patterns of ToBRFV suitable areas under different climate scenarios. This figure was prepared based on the standard map with approval number GS(2020)4619, obtained from the Standard Map Service of the Ministry of Natural Resources of the People’s Republic of China. The base map was not modified. The map is used only for scientific illustration, and the boundary representation does not imply any political or administrative position of the authors or publisher.

**Table 1:** The 21 environmental variables used in this study.

Variable Type	Variable Name (Code)	Unit
Climatic variables	Annual mean temperature (bio1)	°C
	Mean diurnal range (bio2)	°C
	Isothermality (bio3) ^1^	-
	Temperature seasonality (bio4)	%
	Maximum temperature of the warmest month (bio5) ^1^	°C
	Minimum temperature of the coldest month (bio6) ^1^	°C
	Temperature annual range (bio7)	°C
	Mean temperature of the wettest quarter (bio8) ^1^	°C
	Mean temperature of the driest quarter (bio9)	°C
	Mean temperature of the warmest quarter (bio10)	°C
	Mean temperature of the coldest quarter (bio11)	°C
	Annual precipitation (bio12)	mm
	Precipitation of the wettest month (bio13) ^1^	mm
	Precipitation of the driest month (bio14)	mm
	Precipitation seasonality (bio15) ^1^	%
	Precipitation of the wettest quarter (bio16)	mm
	Precipitation of the driest quarter (bio17)	mm
	Precipitation of the warmest quarter (bio18)	mm
	Precipitation of the coldest quarter (bio19)	mm
Agricultural production/facility variables	Tomato harvested area (tomato_host)	ha
	Greenhouse-facility coverage fraction (greenhouse_facility_fraction) ^1^	-

Note: ^1^ indicates variables retained after Pearson correlation and VIF screening for final modeling. tomato_host is coded as bio20 and greenhouse_facility_fraction as bio21.

**Table 2:** Pearson correlation coefficients (r) and VIF values of variables retained in the final model.

Variable	VIF	Bio3	Bio5	Bio6	Bio8	Bio13	Bio15	Greenhouse_Facility_Fraction
bio3	2.263	1						
bio5	2.662	−0.541	1					
bio6	7.186	0.048	0.357	1				
bio8	1.565	−0.152	0.115	−0.078	1			
bio13	5.964	−0.129	0.286	0.605	0.189	1		
bio15	4.882	0.364	−0.430	−0.383	0.441	0.030	1	
greenhouse_facility_fraction	3.718	−0.020	0.352	0.329	0.158	−0.169	0.047	1

Note: Pearson correlation coefficients are shown as a lower triangular matrix; greenhouse_facility_fraction represents the proportion of greenhouse-facility coverage.

**Table 3:** Changes in ToBRFV suitable area under different climate scenarios.

Scenario	Period	Total Suitable Area (×10^4^ km^2^)	Contraction Area (×10^4^ km^2^)	Expansion Area (×10^4^ km^2^)	Stable Area (×10^4^ km^2^)	Loss Rate (%)	Expansion Rate (%)	Overall Range-Change Rate (%)
Current	Current	44.35						
SSP126	2050s	53.84	12.29	21.78	32.06	27.72	49.11	21.39
SSP126	2090s	53.01	7.63	16.29	36.72	17.20	36.72	19.52
SSP245	2050s	44.27	12.17	12.10	32.18	27.45	27.27	−0.18
SSP245	2090s	44.63	17.43	17.71	26.92	39.30	39.93	0.62
SSP370	2050s	43.08	15.34	14.07	29.01	34.58	31.73	−2.85
SSP370	2090s	50.16	16.09	21.90	28.26	36.27	49.38	13.10
SSP585	2050s	27.51	25.10	8.26	19.25	56.60	18.63	−37.97
SSP585	2090s	25.42	29.88	10.95	14.47	67.38	24.69	−42.69

Note: 2050s represents 2041–2060, and 2090s represents 2081–2100.

## Data Availability

The occurrence data and environmental datasets used in this study were obtained from public databases and sources cited in the manuscript. The processed occurrence records, predictor metadata, model configuration, threshold definitions, area-change formulas, and analysis outputs are available from the corresponding author upon reasonable request. Analysis scripts will be deposited in a public repository upon acceptance.

## Supplementary Materials

The following supporting information can be downloaded at https://www.mdpi.com/article/10.3390/biology15191743/s1: Supplementary Table S1. Binary-threshold sensitivity analysis. Supplementary Table S2. Matched Pearson cutoff sensitivity analysis. Supplementary Table S3. Verification of the AUCroc threshold used for the primary area analysis.
