# Supplementary material for: Predicting the Potential Suitable Distribution of Tomato Brown Rugose Fruit Virus in China Using a BIOMOD2 Ensemble Model
Source: Biology (Basel). 2026 Oct 2;15(19):1743. doi: 10.3390/biology15191743 (PMC13663426; doi:10.3390/biology15191743)
Supplement: Supplementary file 1 [file biology-15-01743-s001.zip › biology-4506839-supplementary.pdf]

Supplementary Material

Revision round 2 — Predicting the Potential Suitable Distribution of Tomato Brown Rugose Fruit Virus in China Using a BIOMOD2 Ensemble Model (biology-4506839).

Supplementary Table S1. Binary-threshold sensitivity analysis

Sensitivity of the current and future suitable-area estimates to the binary classification threshold. Each current and future ensemble raster was reclassified at eight thresholds on the 0–1 suitability scale (0.391–0.515, equivalent to 391–515 on the BIOMOD2 0–1000 scale). Areas are reported in 10^4 km² on the valid China-mask grid (24,287 valid cells); NoData cells were excluded from all area calculations.

Threshold (0-1)	Threshold (BIOMOD2 0-1000)	Current suitable area (×10^4 km²)	Scenario	Period	Future suitable area (×10^4 km²)	Contraction area (×10^4 km²)	Expansion area (×10^4 km²)	Stable area (×10^4 km²)	Loss rate (%)	Expansion rate (%)	Signed net range-change rate (%)	Valid current-grid cells (NoData excluded)
0.391	391	52.097	SSP126	2050s	81.9006	11.0281	40.8317	41.0689	21.1684	78.3763	57.2079	24287
0.391	391	52.097	SSP126	2090s	82.7702	6.2058	36.879	45.8912	11.912	70.7891	58.8771	24287
0.391	391	52.097	SSP245	2050s	78.8175	8.2217	34.9422	43.8753	15.7815	67.0713	51.2898	24287
0.391	391	52.097	SSP245	2090s	76.1296	13.6765	37.7091	38.4206	26.2519	72.3824	46.1305	24287
0.391	391	52.097	SSP370	2050s	67.8684	15.139	30.9104	36.958	29.0592	59.3323	30.2731	24287
0.391	391	52.097	SSP370	2090s	93.1264	13.4393	54.4686	38.6577	25.7967	104.5524	78.7557	24287
0.391	391	52.097	SSP585	2050s	63.5599	21.1471	32.61	30.9499	40.5918	62.5948	22.003	24287
0.391	391	52.097	SSP585	2090s	74.5881	31.3847	53.8757	20.7123	60.2428	103.4143	43.1715	24287
0.421	421	47.3933	SSP126	2050s	66.4454	12.1349	31.1871	35.2584	25.6047	65.8048	40.2002	24287
0.421	421	47.3933	SSP126	2090s	63.4413	7.945	23.9931	39.4483	16.764	50.6255	33.8616	24287
0.421	421	47.3933	SSP245	2050s	54.7453	11.7791	19.1312	35.6141	24.854	40.367	15.5129	24287
0.421	421	47.3933	SSP245	2090s	56.2869	16.5619	25.4556	30.8313	34.9458	53.7114	18.7656	24287
0.421	421	47.3933	SSP370	2050s	52.018	16.2457	20.8704	31.1475	34.2786	44.0367	9.7581	24287
0.421	421	47.3933	SSP370	2090s	64.7853	15.4947	32.8867	31.8985	32.6939	69.3912	36.6972	24287
0.421	421	47.3933	SSP585	2050s	41.7013	23.3606	17.6687	24.0326	49.2911	37.2811	-12.01	24287
0.421	421	47.3933	SSP585	2090s	40.1993	30.6337	23.4397	16.7596	64.6372	49.4579	-15.1793	24287
0.441	441	44.3497	SSP126	2050s	53.8362	12.293	21.7796	32.0567	27.7184	49.1087	21.3904	24287
0.441	441	44.3497	SSP126	2090s	53.0061	7.6288	16.2853	36.7209	17.2014	36.7201	19.5187	24287
0.441	441	44.3497	SSP245	2050s	44.2706	12.1744	12.0954	32.1752	27.451	27.2727	-0.1783	24287
0.441	441	44.3497	SSP245	2090s	44.6263	17.4315	17.7082	26.9181	39.3048	39.9287	0.6239	24287
0.441	441	44.3497	SSP370	2050s	43.0848	15.3366	14.0717	29.0131	34.5811	31.7291	-2.852	24287
0.441	441	44.3497	SSP370	2090s	50.1602	16.0876	21.8981	28.262	36.2745	49.3761	13.1016	24287
0.441	441	44.3497	SSP585	2050s	27.511	25.0998	8.2612	19.2498	56.5954	18.6275	-37.9679	24287
0.441	441	44.3497	SSP585	2090s	25.4161	29.8827	10.9491	14.467	67.3797	24.6881	-42.6916	24287
0.461	461	41.6223	SSP126	2050s	43.9149	13.3602	15.6528	28.262	32.0988	37.6068	5.5081	24287
0.461	461	41.6223	SSP126	2090s	44.6659	7.6683	10.7119	33.954	18.4236	25.736	7.3124	24287
0.461	461	41.6223	SSP245	2050s	37.9858	12.5697	8.9332	29.0526	30.1994	21.4625	-8.7369	24287
0.461	461	41.6223	SSP245	2090s	31.3056	20.0799	9.7632	21.5424	48.2431	23.4568	-24.7863	24287
0.461	461	41.6223	SSP370	2050s	33.7959	15.7319	7.9055	25.8904	37.7968	18.9934	-18.8034	24287
0.461	461	41.6223	SSP370	2090s	37.1557	18.4197	13.9531	23.2025	44.2545	33.5233	-10.7312	24287
0.461	461	41.6223	SSP585	2050s	19.8427	27.0762	5.2967	14.5461	65.0522	12.7255	-52.3267	24287
0.461	461	41.6223	SSP585	2090s	15.5342	30.9894	4.9014	10.6328	74.4539	11.7759	-62.6781	24287
0.491	491	37.5114	SSP126	2050s	30.5151	15.1785	8.1822	22.3329	40.4636	21.8124	-18.6512	24287
0.491	491	37.5114	SSP126	2090s	35.1793	9.8423	7.5102	27.6691	26.2381	20.0211	-6.2171	24287

0.491	491	37.5114	SSP245	2050s	29.0921	15.1785	6.7592	22.3329	40.4636	18.019	-22.4447	24287
0.491	491	37.5114	SSP245	2090s	17.2339	24.1117	3.8341	13.3998	64.2782	10.2213	-54.0569	24287
0.491	491	37.5114	SSP370	2050s	25.0208	17.3525	4.8619	20.1589	46.2592	12.961	-33.2982	24287
0.491	491	37.5114	SSP370	2090s	18.6569	24.7441	5.8896	12.7673	65.9642	15.7007	-50.2634	24287
0.491	491	37.5114	SSP585	2050s	13.6765	27.1948	3.3598	10.3166	72.4974	8.9568	-63.5406	24287
0.491	491	37.5114	SSP585	2090s	8.1426	32.2938	2.925	5.2176	86.0906	7.7977	-78.2929	24287
0.500	500	36.6023	SSP126	2050s	28.3016	15.5342	7.2335	21.0681	42.4406	19.7624	-22.6782	24287
0.500	500	36.6023	SSP126	2090s	33.0448	10.2376	6.6801	26.3647	27.9698	18.2505	-9.7192	24287
0.500	500	36.6023	SSP245	2050s	26.0485	16.2062	5.6524	20.3961	44.2765	15.4428	-28.8337	24287
0.500	500	36.6023	SSP245	2090s	15.0204	24.7836	3.2017	11.8187	67.7106	8.7473	-58.9633	24287
0.500	500	36.6023	SSP370	2050s	23.3211	17.5897	4.3085	19.0126	48.0562	11.7711	-36.2851	24287
0.500	500	36.6023	SSP370	2090s	15.7319	25.4556	4.5852	11.1467	69.5464	12.527	-57.0194	24287
0.500	500	36.6023	SSP585	2050s	11.8582	27.5505	2.8064	9.0518	75.27	7.6674	-67.6026	24287
0.500	500	36.6023	SSP585	2090s	7.4311	31.9776	2.8064	4.6247	87.365	7.6674	-79.6976	24287
0.510	510	35.3374	SSP126	2050s	25.7323	16.0086	6.4034	19.3289	45.302	18.1208	-27.1812	24287
0.510	510	35.3374	SSP126	2090s	30.2779	11.0676	6.0082	24.2698	31.3199	17.0022	-14.3177	24287
0.510	510	35.3374	SSP245	2050s	23.9536	16.641	5.2571	18.6964	47.0917	14.877	-32.2148	24287
0.510	510	35.3374	SSP245	2090s	13.1231	25.1394	2.925	10.198	71.1409	8.2774	-62.8635	24287
0.510	510	35.3374	SSP370	2050s	21.2262	17.9454	3.8341	17.392	50.783	10.8501	-39.9329	24287
0.510	510	35.3374	SSP370	2090s	13.7555	25.4556	3.8737	9.8818	72.0358	10.962	-61.0738	24287
0.510	510	35.3374	SSP585	2050s	10.4747	27.511	2.6483	7.8264	77.8523	7.4944	-70.3579	24287
0.510	510	35.3374	SSP585	2090s	6.9963	31.3056	2.9645	4.0318	88.5906	8.3893	-80.2013	24287
0.515	515	34.784	SSP126	2050s	24.6255	16.2457	6.0872	18.5383	46.7045	17.5	-29.2045	24287
0.515	515	34.784	SSP126	2090s	29.1316	11.3048	5.6524	23.4792	32.5	16.25	-16.25	24287
0.515	515	34.784	SSP245	2050s	23.3211	16.641	5.1781	18.143	47.8409	14.8864	-32.9545	24287
0.515	515	34.784	SSP245	2090s	12.2535	25.1789	2.6483	9.6051	72.3864	7.6136	-64.7727	24287
0.515	515	34.784	SSP370	2050s	20.6333	17.8268	3.676	16.9572	51.25	10.5682	-40.6818	24287
0.515	515	34.784	SSP370	2090s	12.8464	25.4951	3.5575	9.2889	73.2955	10.2273	-63.0682	24287
0.515	515	34.784	SSP585	2050s	9.6842	27.5901	2.4902	7.194	79.3182	7.1591	-72.1591	24287
0.515	515	34.784	SSP585	2090s	6.5615	31.0685	2.846	3.7156	89.3182	8.1818	-81.1364	24287

Table S1 note: Loss rate, expansion rate and signed net range-change rate are expressed as percentages of the current suitable area at the same threshold. "Valid current-grid cells" counts only cells with data in both the current and the future raster (NoData excluded).

Supplementary Table S2. Matched Pearson-cutoff sensitivity analysis

Effect of the predictor-screening Pearson cutoff on ensemble performance and on the resulting suitable-area estimates. Two controlled reruns were performed with the VIF cutoff held at <10 and only the Pearson cutoff varied (|r| < 0.7 and |r| < 0.8), using the China-mask domain.

Table S2a. Predictor screening and ensemble performance.

Pearson_cutoff	VIF_cutoff	Pearson_retained_predictors	Final_VIF_retained_predictors	Ensemble_AUCroc_cutoff_0_1000	Ensemble_AUCroc_cutoff_0_1	Current_suitable_area_x1e4_km2	Valid_current_cells	Binary_map_agreement_pct
$\lvert r\rvert < 0.7$	VIF < 10	bio3; bio5; bio6; bio8; bio13; bio15; bio21	bio3; bio5; bio6; bio8; bio13; bio15; bio21	546	0.546	44.7053979495203	24287	97.8795240251987
$\lvert r\rvert < 0.8$	VIF < 10	bio3; bio5; bio6; bio8; bio13; bio14; bio15; bio21	bio3; bio5; bio6; bio8; bio13; bio15; bio21	520.5	0.5205	63.5599291802199	24287	97.8795240251987

Table S2b. Current and future suitable-area change under each Pearson cutoff.

Pearson_cutoff	VIF_cutoff	Scenario	Period	Current_suitable_area_x1e4_km2	Future_suitable_area_x1e4_km2	Contraction_area_x1e4_km2	Expansion_area_x1e4_km2	Stable_area_x1e4_km2	Loss_rate_pct	Gain_rate_pct	Signed_net_range_change_pct	Valid_cells
$\lvert r\rvert < 0.7$	VIF < 10	SSP126	2050s	44.7053979495203	37.9857536953926	22.7282085066085	16.0085642524808	21.9771894429118	50.8399646330681	35.8090185676393	-15.0309460654288	24287
$\lvert r\rvert < 0.7$	VIF < 10	SSP126	2090s	44.7053979495203	34.8630954831803	18.5383126775641	8.69601021122411	26.1670852719562	41.4677276746242	19.4518125552608	-22.0159151193634	24287
$\lvert r\rvert < 0.7$	VIF < 10	SSP245	2050s	44.7053979495203	33.3215300366451	25.0603203359822	13.676452423107	19.6450776135381	56.0565870910699	30.5923961096375	-25.4641909814324	24287
$\lvert r\rvert < 0.7$	VIF < 10	SSP245	2090s	44.7053979495203	30.4360357392844	32.4914563346646	18.2220941244287	12.2139416148557	72.6790450928382	40.7603890362511	-31.9186560565871	24287
$\lvert r\rvert < 0.7$	VIF < 10	SSP370	2050s	44.7053979495203	27.4714868036398	24.7441017828468	7.51019063696628	19.9612961666735	55.3492484526967	16.7992926613616	-38.5499557913351	24287
$\lvert r\rvert < 0.7$	VIF < 10	SSP370	2090s	44.7053979495203	28.3410878247622	34.9816774406061	18.617367315848	9.72372050891423	78.2493368700265	41.6445623342175	-36.604774535809	24287
$\lvert r\rvert < 0.7$	VIF < 10	SSP585	2050s	44.7053979495203	16.0085642524808	36.8394614402767	8.14262774323712	7.86593650924363	82.4049513704686	18.2139699381079	-64.1909814323607	24287
$\lvert r\rvert < 0.7$	VIF < 10	SSP585	2090s	44.7053979495203	16.957219911887	40.8317206736114	13.0835426359781	3.87367727590892	91.3351016799293	29.2661361626879	-62.0689655172414	24287
$\lvert r\rvert < 0.8$	VIF < 10	SSP126	2050s	63.5599291802199	115.301189937003	4.82233293531519	56.5635936920987	58.7375962449047	7.58706467661692	88.9925373134328	81.4054726368159	24287
$\lvert r\rvert < 0.8$	VIF < 10	SSP126	2090s	63.5599291802199	125.183019722485	4.70375097788941	66.3268415201548	58.8561782023305	7.40049751243781	104.353233830846	96.952736318408	24287
$\lvert r\rvert < 0.8$	VIF < 10	SSP245	2050s	63.5599291802199	116.605591468687	5.57335199901182	58.6190142874789	57.9865771812081	8.76865671641791	92.226368159204	83.4577114427861	24287
$\lvert r\rvert < 0.8$	VIF < 10	SSP245	2090s	63.5599291802199	114.905916745584	9.68419318977231	61.0301807551365	53.8757359904476	15.2363184079602	96.0199004975124	80.7835820895522	24287
$\lvert r\rvert < 0.8$	VIF < 10	SSP370	2050s	63.5599291802199	92.5334541112529	11.3048132745913	40.2783382056244	52.2551159056285	17.7860696517413	63.3706467661692	45.5845771144279	24287
$\lvert r\rvert < 0.8$	VIF < 10	SSP370	2090s	63.5599291802199	112.178531724791	10.3166302960432	58.9352328406143	53.2432988841767	16.2313432835821	92.7238805970149	76.4925373134328	24287
$\lvert r\rvert < 0.8$	VIF < 10	SSP585	2050s	63.5599291802199	94.154074196072	13.0835426359781	43.6776876518302	50.4763865442418	20.5845771144279	68.7189054726368	48.134328358209	24287
$\lvert r\rvert < 0.8$	VIF < 10	SSP585	2090s	63.5599291802199	102.533865854161	15.7714003376292	54.74533701157	47.7885288425907	24.8134328358209	86.1318407960199	61.318407960199	24287

Table S2 note: The two reruns used identical settings—the same 35 occurrence records, 21 candidate predictors, pseudo-absence design, cross-validation, algorithms, ensemble settings, and random seed—and differed only in the Pearson cutoff. They were fitted on the same 21-layer predictor stack; because this stack was not numerically identical to the original raster set, the |r| < 0.8 rerun serves as a matched comparator rather than a reproduction of the original model.

Abbreviations: AUCroc, area under the receiver operating characteristic curve; VIF, variance inflation factor; SSP, shared socioeconomic pathway.

Supplementary Table S3. Verification of the AUCroc threshold used for the primary area analysis

This table documents the threshold verification for the original BIOMOD2 ensemble used for the primary current and future suitable-area analysis. The original continuous current ensemble prediction was compared with its corresponding AUCroc binary prediction. Prediction values were stored on the BIOMOD2 0–1000 scale and are also reported on the 0–1 suitability scale.

Evaluation metric	Threshold on the BIOMOD2 0–1000 scale	Threshold on the 0–1 scale	Sensitivity	Specificity
AUCroc	441	0.441	94.444	95.472

Table note: The ensemble evaluation reported an AUCroc threshold of 441. This value was used to define the primary binary suitable/unsuitable classification in the main analysis.

Threshold reproduction from the original binary prediction

Tested threshold (0–1000)	Equivalent threshold (0–1)	Number of discordant cells relative to the archived AUCroc binary prediction	Result
440	0.440	7	Not reproduced
441	0.441	0	Exactly reproduced

Table note: Across the valid China-mask grid, 440 was the highest continuous prediction value classified as unsuitable and 441 was the lowest value classified as suitable. The threshold of 441 exactly reproduced the original AUCroc binary prediction. Accordingly, the primary area analysis in the manuscript uses 0.441 (441/1000).

Abbreviations: AUCroc, area under the receiver operating characteristic curve; BIOMOD2, Biomod platform for ensemble forecasting of species distributions.
